# Three-Dimensional Myoarchitecture of the Lower Esophageal Sphincter and Esophageal Hiatus Using Optical Sectioning Microscopy

**DOI:** 10.1038/s41598-017-13342-y

**Published:** 2017-10-13

**Authors:** Ali Zifan, Dushyant Kumar, Leo K. Cheng, Ravinder K. Mittal

**Affiliations:** 1 Department of Medicine, Division of Gastroenterology, University of California, La Jolla, CA USA; 2 Auckland Bioengineering Institute, University of Auckland, Auckland, New Zealand

## Abstract

Studies to date have failed to reveal the anatomical counterpart of the lower esophageal sphincter (LES). We assessed the LES and esophageal hiatus morphology using a block containing the human LES and crural diaphragm, serially sectioned at 50 μm intervals and imaged at 8.2 μm/pixel resolution. A 3D reconstruction of the tissue block was reconstructed in which each of the 652 cross sectional images were also segmented to identify the boundaries of longitudinal (LM) and circular muscle (CM) layers. The CM fascicles on the ventral surface of LES are arranged in a helical/spiral fashion. On the other hand, the CM fascicles from the two sides cross midline on dorsal surface and continue as sling/oblique muscle on the stomach. Some of the LM fascicles of the esophagus leave the esophagus to enter into the crural diaphragm and the remainder terminate into the sling fibers of the stomach. The muscle fascicles of the right crus of diaphragm which form the esophageal hiatus are arranged like a “noose” around the esophagus. We propose that circumferential squeeze of the LES and crural diaphragm is generated by a unique myo-architectural design, each of which forms a “noose” around the esophagus.

## Introduction

With advances in the manometry techniques over the last 50 years, the functional existence of the smooth muscle lower esophageal sphincter (LES), as an “intrinsic LES” and skeletal muscle crural diaphragm (CD) as an “extrinsic LES” at the esophago-gastric junction (EGJ) in humans is well accepted. The esophageal hiatus formed by the two crura was originally thought to be the major anti-reflux barrier^1^. However, with the invention of water perfused manometry technique by Harris *et al*.^2^, and a series of publications^3–6^ in the 1960’s and 1970’s proved the significance of smooth muscle LES in reflux disease, and esophageal motor disorders. Studies by Boyle *et al*.^7^ in early 1980’s, and later on by our laboratory^8,9^ proved beyond doubt that the CD is an important component of the sphincter mechanism at the EGJ, and it is now called as the “external LES”^10^, analogous to the internal smooth muscle and external skeletal muscle anal sphincters at the caudal of gut.

In spite of all the advances mentioned above, and proof of the functional existence of the LES and CD, their anatomical/structural morphology remains controversial. Debate continues whether the LES is only a functional entity; the controversy stems from the observation that the investigators have not found an expected annular ring of thickened muscle in the region of the LES. Not surprising therefore, that Ingelfinger (editor of New England Journal of Medicine) called the LES a “sphinx”^11^. The Most widely quoted publication of the LES morphology is based on the work of Liebermann-Meffert *et al*.^12^, in which they described a semicircular clasp (on the lesser curvature) and oblique sling fibers (towards the greater curvature), with clasp fibers inserted into the sling fibers^13^.

In 2010, Yassi *et al*.^14^ made a novel attempt to study the microscopic myoarchitecture of the LES and CD. However, lack of a powerful, central and graphical processing unit impeded the reconstruction of the 3D anatomy of the EGJ at the micro resolution. In this follow up study, we use the same specimen to report on an actual 3D reconstruction of the myoarchitecture of the EGJ at the microscopic level. It should be emphasized that most depictions of the EGJ in the literature are either schematics or drawings of the surgical dissection of the LES.

## Methods

The details of image acquisition are described in the publication of Yassi *et al*.^14^. Ethics approval was obtained from the Northern Regional Ethics Committee (Auckland District Health Board, New Zealand) for the en-bloc excision of the lower esophagus, GOJ, upper stomach, and central diaphragm from a fresh cadaver at postmortem. Informed consent was obtained by the staff at the mortuary at Auckland Hospital from the mother of a 19-year-old male. All experiments were performed in accordance with relevant guidelines and regulations of the University of Auckland, New Zealand. The tissue was excised 10 hours after death. There was no history of esophageal symptoms, such as gastro-esophageal reflux or dysphagia, and prior surgery to the region. A 1.5 cm length of the distal esophagus, 3 cm length of cardia of the stomach along with a 2 cm circumferential cuff of the diaphragm from a cadaver was harvested in en-bloc. The specimen was soaked in 3% buffered formalin solution for seven days and then dehydrated in a graduated series of ethanol concentrations. The dehydrated specimen was then embedded in wax using a TISSUE-TEK VIP 2000 automatic processor (Global Medical Instrumentation, MN).

A custom-built, extended-volume imaging system^15^ was used to obtain a 2D images of the tissue block at the 8.2 µm/pixel resolution. The system consisted of a high-precision (0.1 µm step) three-axis (XYZ) Aerotech translation stage mounted on a Newport anti-vibration table (Newport Corporation, Irvine, CA), an imaging facility [Leica 4D confocal microscope and an 8.2 megapixels digital camera (Canon 1D mark II, Tokyo, Japan) with a Canon 65-mm Photomicro lens and a tissue milling facility (Leica SP2600 ultramill, Nussloch, Germany).

The surface of the tissue block was stained for few seconds with May–Grunwald stain to distinguish different histological layers through the wall of the specimen, muscle layers (green) and connective tissue (pink/purple) and imaged, following which the stained surface was removed by the ultramill. The milling, staining, and imaging cycle were repeated at 50 µm in the Z direction throughout the tissue block. Because of the large size of the tissue block, to capture the entire X–Y (in-plane in relation to the stage) surface of the sample, the system was setup to capture nine sub images within a 3 × 3 × 3 matrix. The sub images were acquired in RAW format, transferred to the control computer, and stored in an indexed file structure. The final stack of 652 montage images was cropped to enclose the tissue region, resulting in an image volume of 7,000 × 5,816 × 650 pixels (57.5 × 47.8 × 32.45 mm^3^).

### Image Processing

**Figure 1:**
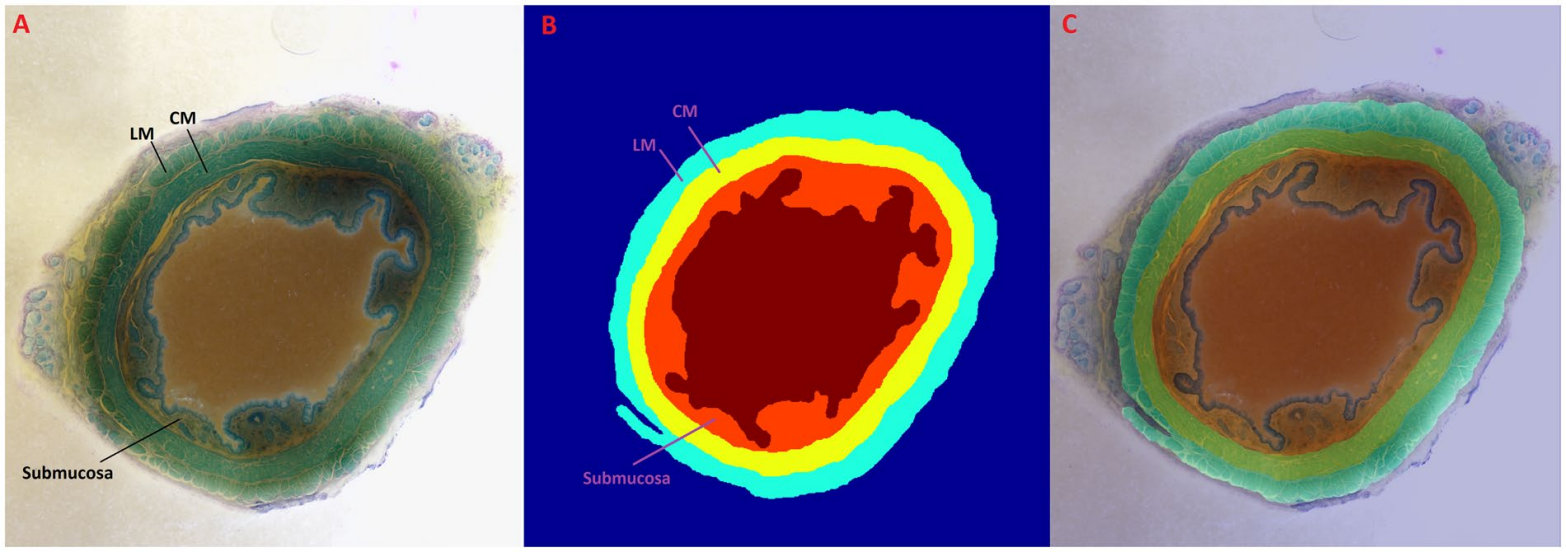
A cross sectional image from the cranial end of the specimen. (**A**) It shows various layers of the esophageal wall. (**B**) Shows segmentation of the circular and longitudinal muscle layers alongside the superimposed image in (**C**).

All 652 cross sectional images were first imported into the AMIRA (Visage, Carlsbad, California). The outer margin of the longitudinal muscle layer, inner and outer margins of the circular muscle were manually marked and outlined as shown in Fig. 1, and imported into Matlab (The Mathworks, Inc.) to build a 3D structure of the entire specimen using the most powerful graphical processing unit currently available in the commercial market (Dell Precision T7910, Dual Intel Xeon Processor E5-2687W v4, NVIDIA Quadro M6000 24GB, 256GB RAM).

## Results

**Figure 2:**
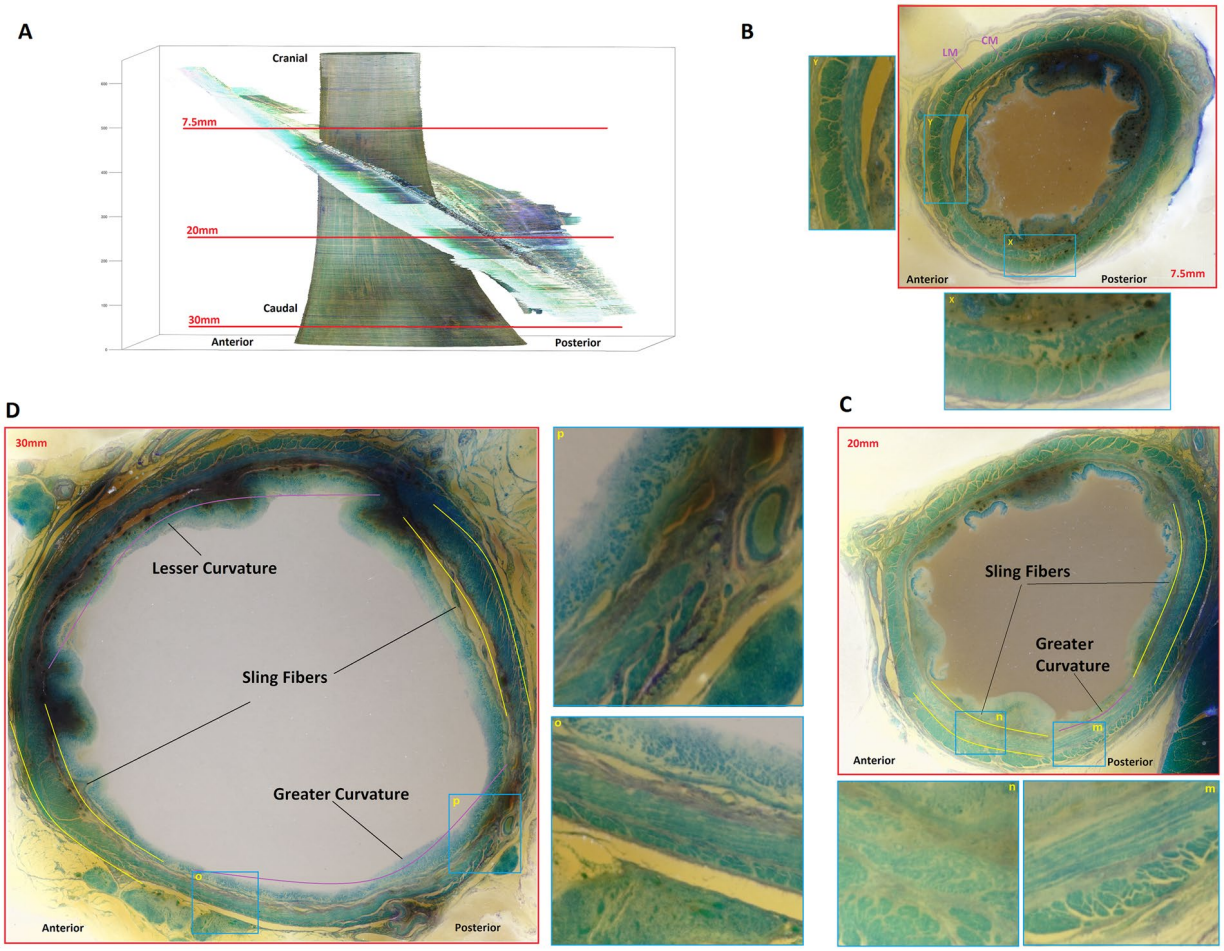
Axial sections of the esophagus at the mid and lower levels of the specimen. (**A**) Three-dimensional reconstruction of the segmented layers. (**B**) The arrangement of muscle fibers shows different shapes of muscle fibers along different part of the circumference, left anterior part of the circumference (x = magnified inset) shows muscle fibers of different shapes and size suggesting crossing of fibers. On the other hand, the remainder of the circumference shows fibers in the long axis of the circular muscles (y = magnified inset). (**C**) Shows two area of muscle thickening around the circumference where fibers are arranged in an oblique fashion (marked by yellow lines). Also note circular muscle fibers in between two oblique bundles along the lesser and greater curvature side of the stomach are inserted into the oblique muscle layer. (**D**) Shows that the two areas of increased muscle thickness move in the circular muscle circumference, towards the lesser curvature. Also note that the longitudinal muscle layer is thicker towards the lesser curvature as compared to the greater curvature side.

We identified 3 different arrangements of muscle fibers in the circular muscle layers of the specimen in Fig. 2. Arrangement 1 consisted of muscle fibers in their long axis around the entire circumference of the specimen, see Fig. 1A (7.5 mm from the cranial end). Arrangement 2: the circular muscle fibers in the left anterior part of the specimen revealed a crisscross pattern (7.5 mm, to 20 mm), which occupied approximately 25% of the circumference of the esophagus, the remainder 75% shows circular muscles in their long axis (Fig. 2B, slice at 7.5 mm from the cranial end). Arrangement 3 consisted of two distinct regions of increased muscle thickness located in the circular muscle layer (Fig. 2C, slice at 20mm from the cranial end). The muscle fibers in these two regions of increased thickness are arranged in an oblique fashion, unlike the rest of the circumference where the fibers are arranged in their long axis (circular muscle layer). These two oblique muscle bundles are close to one another on the left side of the specimen at the cranial end and moved apart in the circumference, from the cranial to caudal end of specimen (Fig. 2D, slice at 30mm from the cranial end).

**Figure 3:**
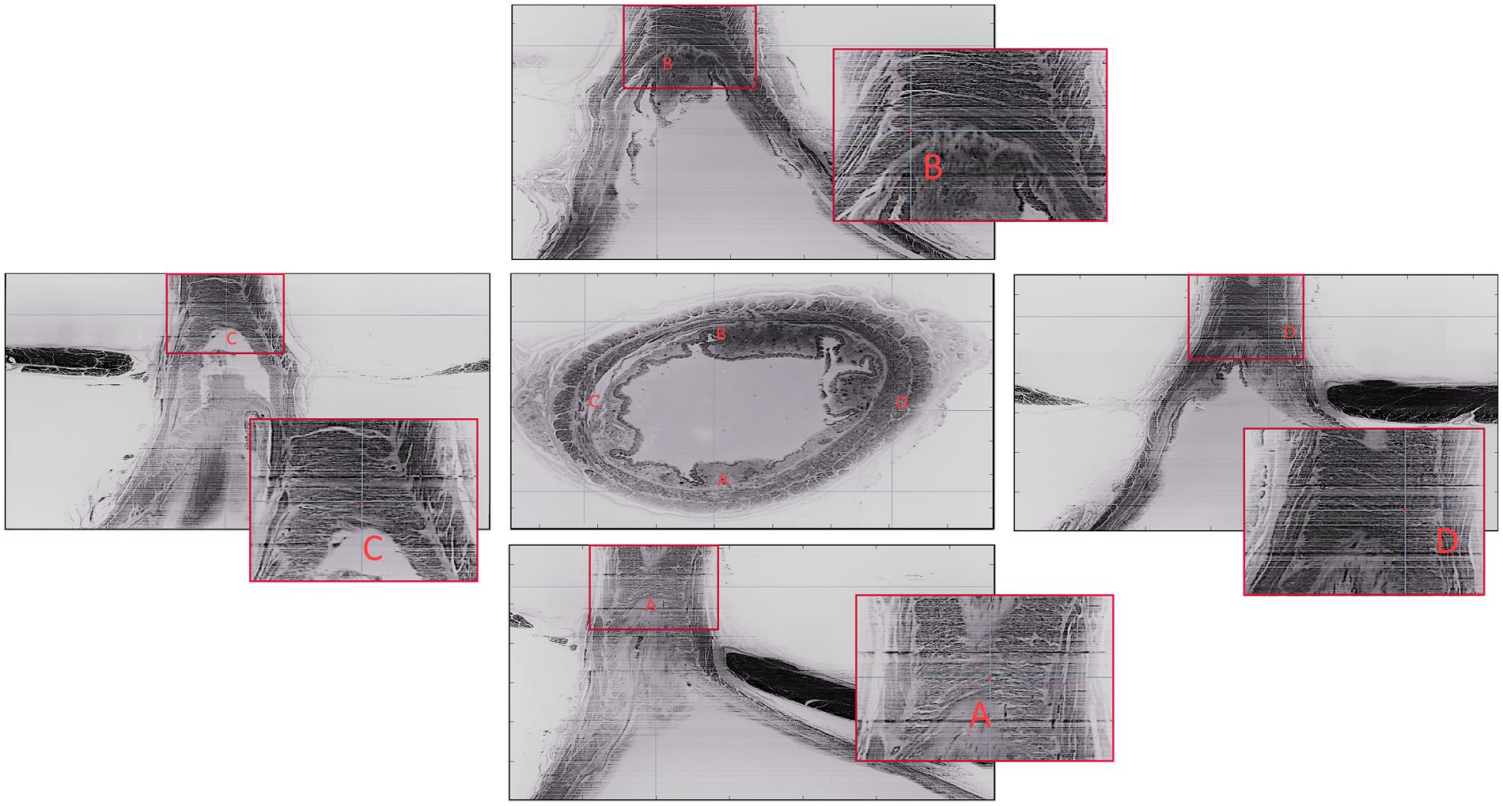
Coronal sections along the ventral (**B**), dorsal (**A**) and sagittal sections the left (**C**) and right (**D**) walls of the esophagus through the circular muscle layer. Note spiral arrangement of the muscle fascicles on the ventral and lateral surfaces of the circular muscles. On the other hand, note crossing of circular muscle fibers on the dorsal surface.

Figure 3 shows coronal sections through the circular muscle layer along the ventral, dorsal, right lateral and left lateral walls of the upper half of the specimen. On the ventral and lateral walls, the muscle fibers are arranged in spiral fashion with a slant downwards from the right to left. On the other hand, the muscle bundles show a pattern of crossing from one side to the other in the middle of the specimen on the dorsal wall (point A). Note, the crossing of muscle bundles in the middle of the specimen and after their crossing they continue as the oblique muscle bundles in the dorsal wall.

**Figure 4:**
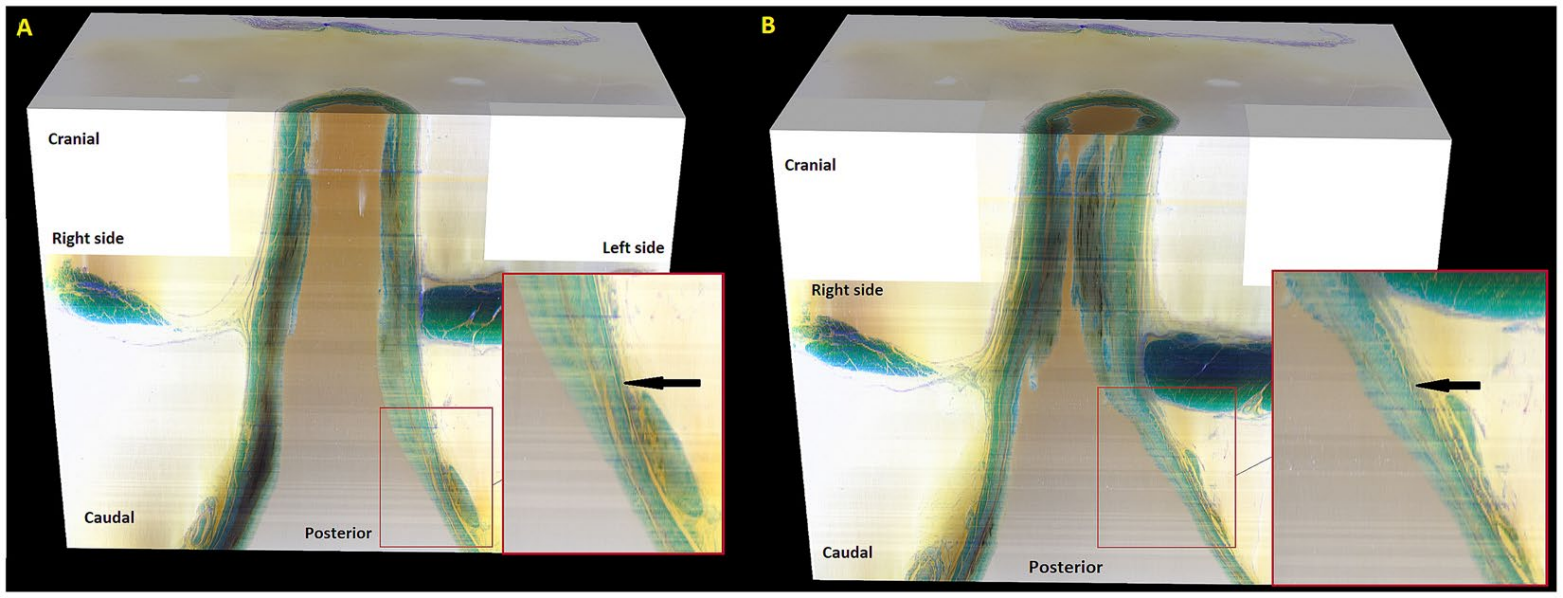
Serial coronal sections through the lumen the anterior esophagus (**A**) and posterior (**B**) aspect. Note how the longitudinal muscle fibers on the left side (towards the greater curvature) terminate into the circular/oblique muscle fibers but not the right (towards the lesser curvature).

Coronal sections of the specimen through the lumen of the esophagus from the anterior to posterior direction reveal that some longitudinal muscle fibers of the esophagus leave the esophagus and merge into the crural diaphragm (see Fig. 4). The remainder of the longitudinal muscle fibers get thinner and merge into the circular/oblique muscle layer as they approach the caudal end of specimen on the left side (towards the greater curvature of the stomach) (thin arrow) but not on the opposite side, i.e., right side, towards the lesser curvature of the stomach; these fibers continue towards the caudal end of the specimen. The merging of the longitudinal muscle fibers into the oblique muscle bundles can also be seen in the cross sectional or axial images.

**Figure 5:**
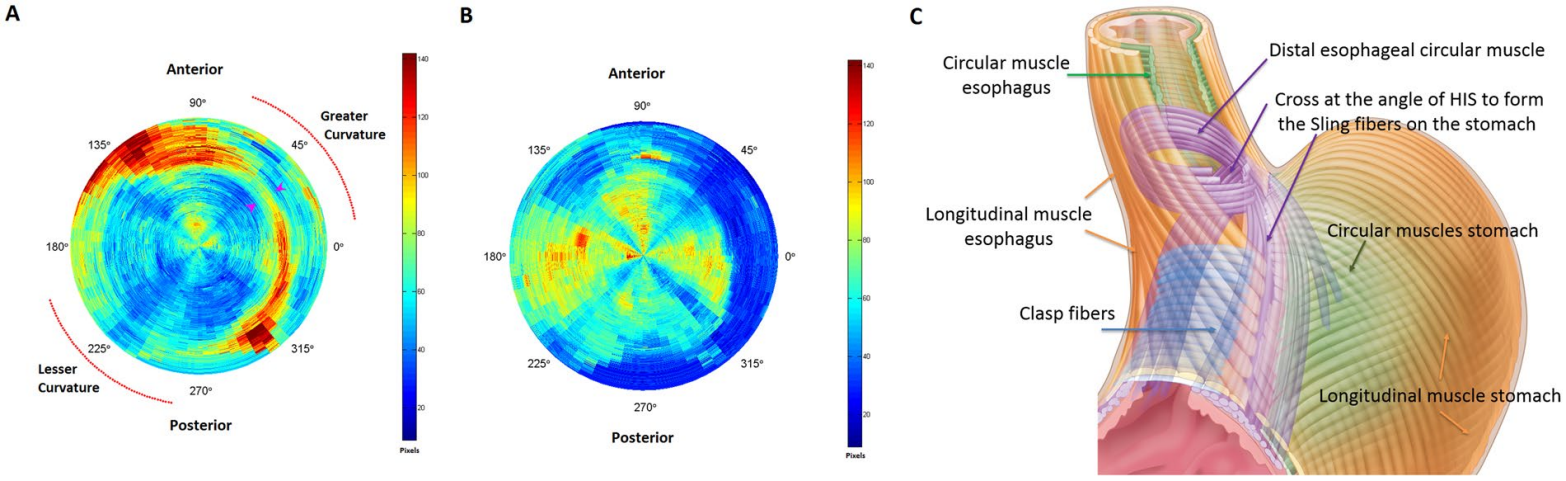
Shows the thickness of the circular (**A**) and longitudinal muscle (**B**) layers along the length of the specimen. The lumen in each of the slices was cut into 64 equally spaced intervals, covering the entire circumference of both the muscle layers. Prior to the angular slicing, as the curvature of the specimen varies in the Z-direction for each slice, the centroid of the lumen was calculated and used as the center of the angular cut. Thickness was calculated as the sum of the number of pixels from the segmented regions falling in each of the 64 intervals. Outer margin of the circle represents the stomach (caudal end of specimen) and the center of circle is the esophagus (cranial end of specimen). Note, that the locations of increased circular muscle thickness in (**A**) at the angle of His on the greater curvature and two oblique/sling muscle bundles as they descend from the greater curvature of the stomach towards the lesser curvature. Also note the longitudinal muscle is thinner on the greater curvature side at the level of the sling fibers of the stomach, (**C**) Schematic of the microscopic myoarchitecture of the circular and longitudinal muscle layers of the lower esophageal sphincter and stomach. The circular muscle fibers of the esophagus cross to the opposite side at the angel of His to continue as the oblique muscle fibers (innermost muscle layer of the stomach) on the ventral and dorsal surface of the stomach.

Figure 5 shows the thickness of the circular and longitudinal muscle layer along the specimen. The outer margin of the circle represents the caudal end of specimen (gastric side) and the inner the cranial end (esophagus). Note, that the two regions of increased circular muscle thickness traversing from left side of the specimen (on the greater curvature) towards the right side (lesser curvature) in the caudal half of the specimen. These two regions of increased muscle thickness are the two oblique muscle bundles described in the cross sectional images. In the caudal part of the specimen the longitudinal muscle is thinner around the greater curvature of the stomach as compared to the lesser curvature. Panel (C) shows a schematic of the myoarchitectural design in the specimen. The circular muscle fibers at the ventral wall of specimen (esophagus) are arranged in a spiral fashion. On the other hand, the circular muscle fibers cross midline from one side to the other at the dorsal surface and then continue as oblique muscle fibers or sling fibers of the stomach/LES. The crossing of muscle fibers is located on the left side, i.e., close to the angle of His, from where the two bundles of oblique muscle fibers move apart as they move from the greater curvature of the stomach towards the lesser curvature on the anterior and posterior wall of the stomach.

### Muscle fiber orientation at the dorsal and ventral sides

**Figure 6:**
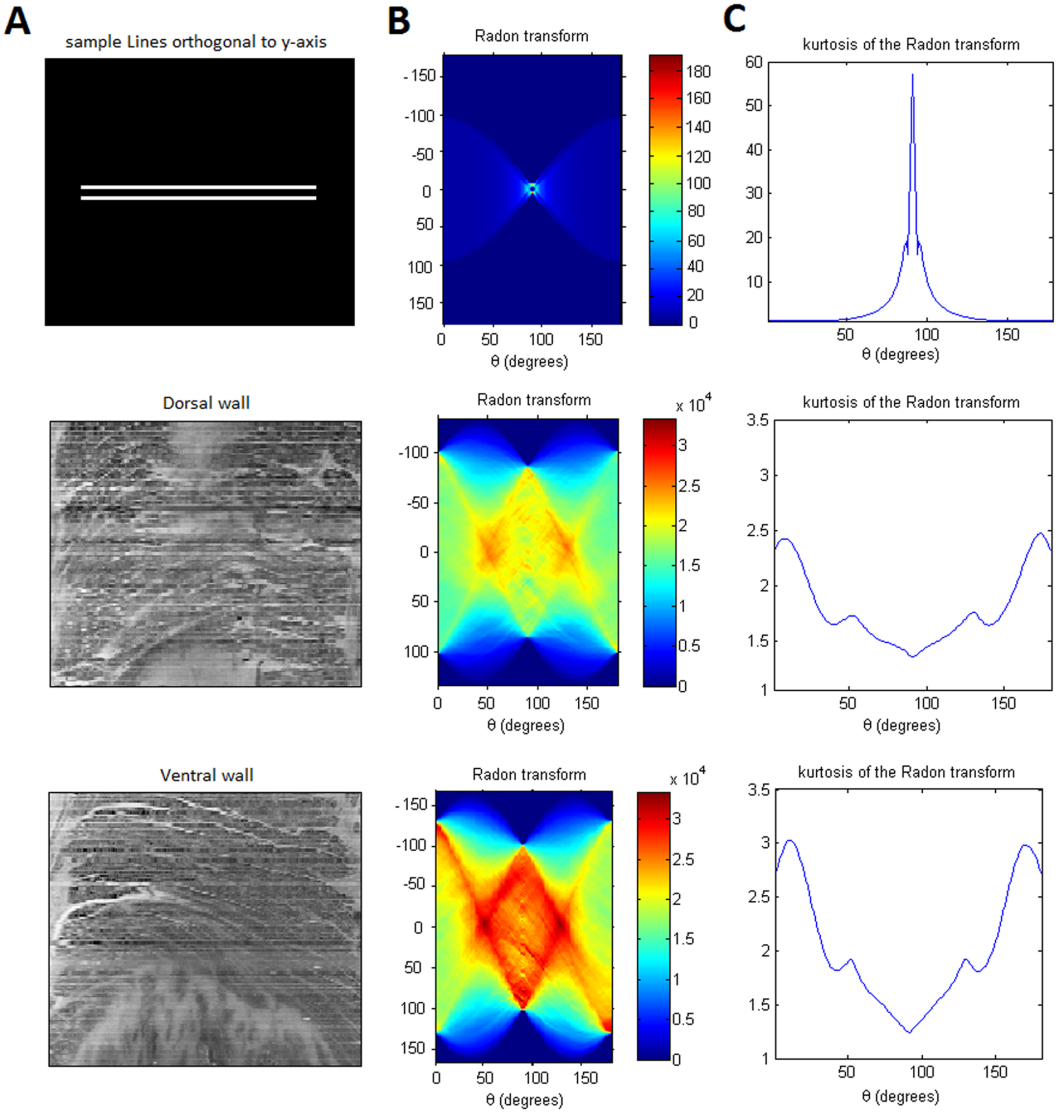
Shows muscle fiber orientation estimation. As shown, the principal direction of the muscle fiber is first estimated by applying the Radon transform, shown in Panel (B), on the original images of Panel (A). Next, calculating the fourth order moment (or Kurtosis) on the former transform, produces the images in Panel (B). For example, for the 2 synthetic parallel lies(top row of panel A), that are both perpendicular to the y-axis, the peak of the Kurtosis happens at 90 degrees, which is in accordance to the original fiber orientation. The same approach is carried out for both the dorsal and ventral sides of the specimen. As it can be seen, the kurtosis shows larger peaks at the ventral side, indication a more oriented set of fiber, compared to the flatter kurtosis distribution at the dorsal side.

The Radon transform used in reconstructing Computed Tomography (CT) slices has also been used to quantify the predominant orientation in a repeated structure, such as the muscle fascicles in an ultrasound image^16^. The Radon transform projects a grid of parallel lines, one pixel apart, across the image and calculates the integral of the image intensities along each line^16^. The orientation *y* of the grid is varied, and when *y* approaches the dominant orientation of structures within the image then the Radon transform has greatest variability across the image: this variability is quantified by its kurtosis, which measures the degree to which a distribution is peaked, and its peaks describe predominant fiber orientations within the image. Since real biological images have discontinuous line-like structures at unequal distances relative to each other (as opposed to uniformly distributed continuous lines in the synthetic images), the measure of kurtosis is more sensitive to fascicle orientation. As it can be seen in Fig. 6 the radon transform is darker and its kurtosis distribution flatter on the dorsal side compared to the ventral side, indicating a more directional distribution of fibers in the ventral side compared to the crossing muscle fibers on the dorsal side.

**Figure 7:**
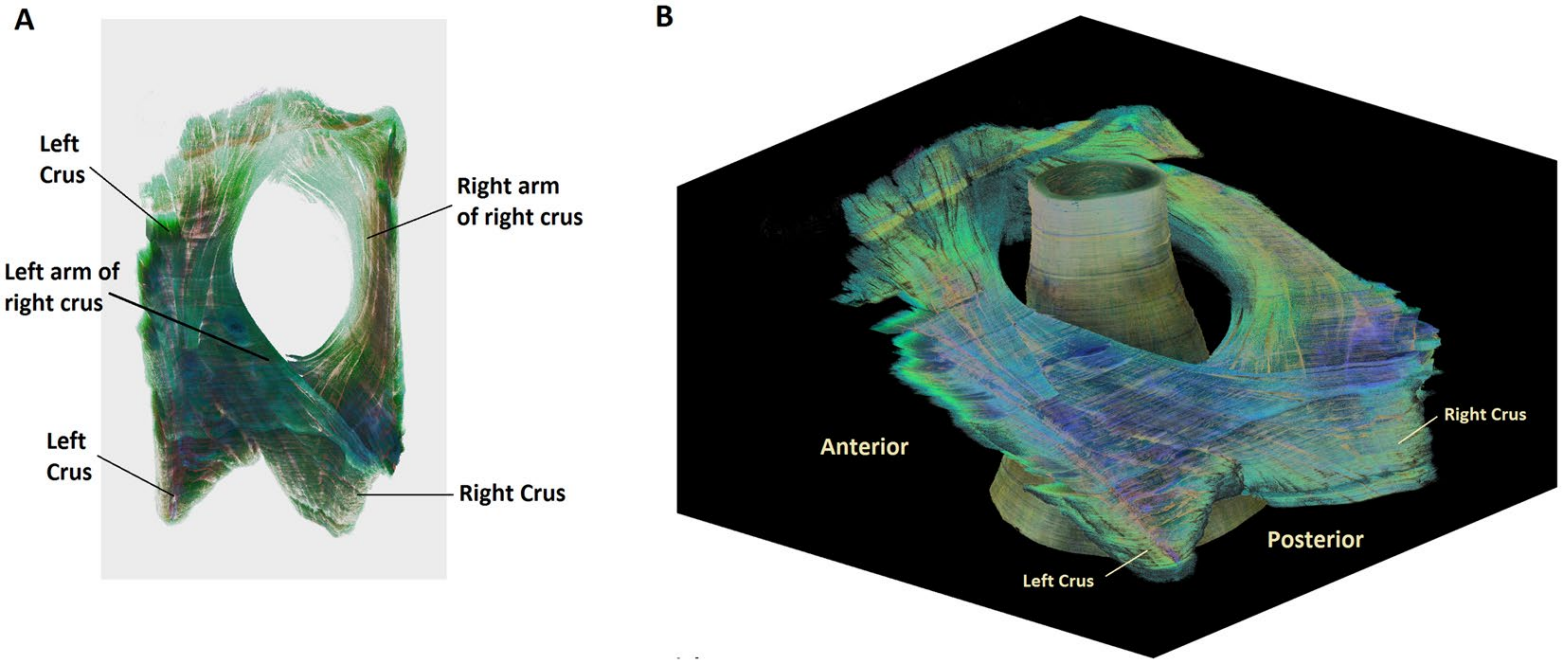
Shows the microscopic morphology of the esophageal hiatus. (**A**) is the superior view of the esophageal hiatus and (**B**) is the posterior view. Note, that the two bundles of right crus cross each other first and then encircle the esophagus to form the esophageal hiatus or in other words, a noose- like arrangement of the muscle fascicles of the right crus. Also note crossing of fibers at the ventral end of the hiatus.

Figure 7 shows the superior (A) and posterior view (B) of the crural diaphragm and esophageal hiatus. The right and left crus are seen clearly in the specimen. The right crus consists of two distinct bundles, they cross each other first and then surround the esophagus. The left crus joins the left bundle of the right crus to augment the left hiatal margin of the esophageal hiatus. The muscle bundles from the right and left hiatal margins cross each other again at the ventral surface of the esophagus, at the cranial end of the specimen.

## Discussion

Our major and novel finding is that the circular muscle fibers at the lower end of the esophagus cross on its dorsal surface, close to the angle of His and continue as oblique muscle fibers on the anterior and posterior surface of the stomach (from the greater curvature to the lesser curvature). In the cranial part of the specimen (esophagus), the circular muscle fibers are arranged in a spiral fashion. In the middle part of the specimen (LES) the muscle fibers cross midline to continue as oblique/sling fibers of the LES. In the caudal part (stomach), there are two bands of oblique muscle fibers on the anterior and posterior surface with circular muscles interposed between the two oblique muscle bands. Our findings are similar to that of Liebermann-Meffert^12^ but only in the caudal part of specimen, i.e., circular muscle fibers from the lesser curvature of the stomach, what authors called clasp fibers merge into the sling/oblique muscle fibers descending from the angle of His towards the greater curvature of the stomach^12^. We propose that the spiral muscle fibers of the distal esophagus, crossing at the angle of His (not described by Liebermann Meffert^12^) are the important components of the LES because one can easily visualize how such an arrangement will provide a circumference squeeze at the lower end of the esophagus. The LES muscle fibers are actually arranged like a “noose” around the esophagus, rather than like a ring or a band of circular muscle fibers. The circular muscle fibers from the greater curvature of the stomach (similar to from the lesser curvature) also merge with the two oblique muscle bands on the anterior and posterior surface of the stomach. Interestingly, using anatomical dissection a description similar to ours was reported by Jackson (1978)^17^. However, he did not study the longitudinal muscle layer. In our specimen the longitudinal muscle fibers on the left side merge into the oblique muscle bundles, close to the angle of His.

Whether the LES is an anatomical/structural or only a functional entity has been debated extensively^14,18^. Some studies found thicker circular muscles on the left side of the distal esophagus, close to the angle of His, towards the greater curvature of the stomach^19^. Ultrasound imaging clearly show that the circular muscle in the LES high pressure zone is thicker than the rest of the esophagus^20,21^. We observed that the only regions of increased circular muscle thickness in our specimen were the regions of fiber crossing at the angle of His and the two oblique muscle fiber bundles located on the anterior and posterior surface of the stomach, as they descend from the angle of His. We propose that it is the architecture/design of the muscle fibers in the LES region that distinguishes the LES muscles from those of the adjacent esophagus. Based on our finding, we propose that a “noose” like arrangement of muscle fibers is the critical hallmark feature of the LES myoarchitecture.

There are two interesting observations regarding the myoarchitecture of the proximal stomach, also called the cardia of the stomach. We found that the circular muscles do not go all around the circumference; they merge with the oblique bundles close to their margins. The two oblique bands begin at the angle of His, from the muscles of distal esophagus (LES) after they cross the midline, on the greater curvature and then move towards the lesser curvature of the stomach. Based on the published schematic drawings, it appears that the “cardiac muscle loop” described by Willis in 1674^22^ and collaris Helvetti described in 1719 by Helvitus^23^ are the same structure as the inner oblique muscle layer of the stomach, which is the same structure as the sling fibers of the LES described by Liebermann-Meffert *et al*.^12^, and more recently by Vegesna *et al*. in micro CT imaging^24^. We find that the sling fibers of the LES originate from the spiral muscle fibers of the distal esophagus which after crossing at the angle of His continue as the sling fibers of the stomach. The sling/oblique muscle bundles are unique structures because the longitudinal muscle fibers of the esophagus terminate into them and the longitudinal muscle fibers of the stomach appear to begin in the oblique muscle layer. Furthermore, the circular muscle fibers of the stomach, from both the greater and lesser curvature of the stomach appear to merge with the oblique muscle bundles on the anterior and posterior surface of the stomach as well.

Esophageal hiatus was considered to be the major sphincter mechanism at the lower end of the esophagus before the discovery of the smooth muscle LES. A study of 204 fresh cadavers by Listerud and Harkins^1^ in 1959 found that the esophageal hiatus is formed predominantly by the right crus of the diaphragm. They described eleven different configurations how right and left crus come together to form the esophageal hiatus. The arrangement that we found in our specimen was the most common one found in their study (49%), i.e., right crus as the major contributor in the formation of esophageal hiatus. The novelty of our study is the arrangement of muscle bundles in the right crus; they are not organized as circular ring, instead they form a “noose” around the esophagus.

Knowledge of the precise myoarchitecture of each sphincter is of fundamental importance in improving understanding of its function, preventing damage and enhancing function. Therefore, our findings have clinical significance, e.g., Heller’s myotomy performed along the angle of His, where muscle fibers from the right and left side cross midline to continue as sling fibers is more likely to disrupt the LES high pressure zone more effectively than the one performed in any other circumferential orientation of the LES. Along those lines, it is interesting that a recent study reported that patients with failed myotomy along the lesser curvature of the stomach succeeded with a repeat myotomy placed along the greater curvature of the stomach^25^.

A limitation of the current study is the analysis of a tissue block from a single individual. However, the logistics, time and effort needed to obtain, process and analyze such data should not be under-estimated (e.g., over 100 hours was required to just stain and image the tissue block producing 75 Gb of raw data, and the subsequent manual delineation and analysis of the images took more than one year). The analysis of high-resolution ‘single sample’ datasets (e.g., Visible Human Project^26^), has provided unique insights into what is traditionally assumed to be standard knowledge of anatomy. Along those line, our findings should raise interest in the studies of the microscopic myoarchitecture of the LES and other sphincters. We envision that diffusion tensor magnetic resonance imaging or micro CT imaging technique, which may be less cumbersome than our methodology, may provide confirmatory evidence of our findings.
